# Editorial: Metabolic diseases and healthy aging: prevention and public health policy based on risk factors

**DOI:** 10.3389/fpubh.2024.1502564

**Published:** 2024-10-22

**Authors:** Mingxin Bai, Xiaodong Sun, Xiao Tan, Yun Gao

**Affiliations:** ^1^Department of Endocrinology and Metabolism, Diabetic Foot Care Center, West China Hospital, Sichuan University, Chengdu, Sichuan, China; ^2^Department of Endocrinology and Metabolism, Affiliated Hospital of Weifang Medical University, Weifang, China; ^3^Department of Neuroscience, Uppsala University, Uppsala, Sweden; ^4^Department of Clinical Neuroscience, Karolinska Institutet, Solna, Sweden

**Keywords:** metabolic diseases, healthy aging, public health, risk factors, prevention strategies

With the rapid global increase in aging populations, healthy aging has become a major challenge to public health worldwide. Healthy aging is defined as the process of maintaining functional ability to enable wellbeing in older age by the World Health Organization (WHO). Aging has been known to significantly increase the susceptibility of older adults to age-related diseases, including metabolic disorders. Additionally, several metabolic diseases, including diabetes, hypertension, and their complications (cardiovascular and renal diseases et al.) impose significant barriers to healthy aging. The development of these metabolic diseases can be attributed to certain risk factors, including smoking, poor dietary habits, obesity and a sedentary lifestyle. Moreover, these risk factors can also affect the aging process itself, by disrupting the balance of metabolic regulation in the body. Prompt and appropriate interventions targeting these risk factors can mitigate the impact of metabolic diseases and promote healthy aging. Therefore, understanding the complex interactions between aging and metabolic diseases is essential for improving public health outcomes in older populations.

This Research Topic focuses on exploring the associations between metabolic risk factors, chronic and metabolic diseases, and healthy aging. We wish to unveil evidence on how to prevent, treat, and manage metabolic risk factors and diseases in older adults, in order to offer recommendations for future research and policy interventions toward healthy aging and public health.

The Research Topic comprised one literature review article and nine original research articles, primarily focusing on risk factors for healthy aging, metabolic risk factors and diseases in older adults and the current state of metabolic diseases and healthy aging. These articles provide valuable insights into effective public health interventions that can bring beneficial outcomes for healthy aging and chronic metabolic diseases.

Yang, Liu et al. utilized Mendelian Randomization to explore the connection between sarcopenia and digestive system illnesses. Their findings suggested that reduced muscle mass may raise the risk of gastroesophageal reflux disease and non-alcoholic fatty liver through the exacerbation of metabolic disorders.

Mamgai et al. assessed the cardiovascular risk of a large sample from the aging population in India. Their findings indicated that rural, poor, less-educated, and diabetic individuals face higher cardiovascular risks, while regular exercise reduces this risk. Importantly, the study highlighted the need for early detection and management of hypertension, as undiagnosed hypertension poses similar cardiovascular risks.

Chen et al. provided evidence that accelerometer-based physical activity may causally lower the risk of geriatric syndromes, while sedentary behavior may increase the risk of geriatric syndromes (frailty, falls, and dysphagia), emphasizing the significance of strengthening physical activity to improve the quality of life for older adults.

Dąbek et al. conducted a questionnaire survey in Poland, to compare health behaviors between seniors attending and not attending Universities of Third Age (UTA's) classes. They found the positive impact of UTA's classes on seniors' health-promoting behaviors (e.g., physical activity, alcohol consumption, and preventive tests performance).

Wu et al. found that bone mineral density was positively correlated with cardiometabolic index (CMI), suggesting a critical role for lipid metabolism in osteoporosis. CMI may serve as a potential new marker for the diagnosis and prevention of osteoporosis by assessing lipid metabolism levels.

Ravindranath et al. demonstrated patient journeys in primary care for managing hypertension and diabetes in Kerala, India, using Levesque's access framework. They identified several factors influencing access to primary health services for these conditions, highlighting the necessity to enhance timely diagnosis, treatment, and ongoing care within the lower levels of the healthcare system. Furthermore, this study emphasized the necessity of establishing healthcare policies that closely link non-communicable diseases with their social determinants.

Zhou et al. explored the relationship between osteoporosis and multiple special diets through Mendelian Randomization analysis. They identified a significant association between a gluten-free diet and increased osteoporosis risk. Interestingly, the results suggested a hypothesis that in addition to Celiac Disease, a gluten-free diet used for its treatment may also lead to osteoporosis.

Wang et al. revealed the growing demand for “Internet + Traditional Chinese Medicine” home nursing services among older adults with chronic diseases. To provide directed and diversified Chinese medicine home care services, they recommended strengthening demand, improving the service system, and ensuring high-quality care.

Yang, Wang et al. conducted a large-sample study to explore the prevalence and influencing factors of abnormal carotid artery intima-media thickness in Henan Province, China. The results highlighted the significance of early screening for at-risk populations, particularly older men and individuals with hypertension, diabetes, or dyslipidemia.

Zhang et al. conducted an extensive review to explore the complex interaction between environmental and behavioral risk factors with metabolic diseases and their impact on healthy aging. It identified key contributors such as environmental pollutants, diet, physical activity, smoking, alcohol consumption, sleep patterns, and psychological stress, all linked to metabolic disorders and age-related complications. Moreover, they contributed important insights aimed at promoting public health and encouraging healthy aging.

This Research Topic highlights the importance of understanding risk factors associated with metabolic diseases and healthy aging. These findings provide valuable insights into prevention strategies and public health policy to promote healthy aging.

